# Effect of hyaluronic acid on cytokines and immune cells change in patients of knee osteoarthritis

**DOI:** 10.1186/s12891-022-05767-y

**Published:** 2022-08-25

**Authors:** Lixia Jin, Kangli Xu, Yun Liang, Peng Du, Shengcheng Wan, Chang Jiang

**Affiliations:** 1 Department of Rehabilitation Medicine, Zhongshan Hospital, Fudan University, Shanghai, China; 2 Department of Laboratory Medicine, Zhongshan Hospital, Fudan University, Shanghai, China; 3 Department of Orthopedics, Zhongshan Hospital, Fudan University, Shanghai, China

**Keywords:** Knee osteoarthritis, Hyaluronic acid, Macrophages through polarization

## Abstract

**Purpose:**

To evaluate the changes of cytokines and immune cells after Intra-articular hyaluronic acid(IAHA)injections in patients with knee osteoarthritis (KOA).

**Patients and Methods:**

Sixteen patients were included in the study, with a total of 65 IAHA injections. The Numeric Rating Scale (NRS) and Lysholm scores were evaluated at each visit. The immune cells and 14 cytokines of synovial fluid were analyzed at each visit. The association between immune cells and cytokines were examined.

**Results:**

IL-6 and IL-8 were the most common cytokines in the synovial fluid of KOA patients. The synovial fluid was orchestrated by macrophages (69%) and Lymphocytes (18%). Neutrophils were less to count of the total cell population (< 2%). The cytokines decreased significantly after the first injection and then tended to be stable. Lymphocytes increased a lot, while Macrophages decreased in the early stage, then increased after multiple injections. The proposition of M1 decreased in the early stage, then increased after multiple injections, while M2 increased consistently. M1 and M2 were positively associated with IL-6 and IL-8.

**Conclusion:**

The synovial fluid of KOA patients was orchestrated by macrophages (69%) and Lymphocytes (18%) and cytokines like IL-6 and IL-8. IAHA may play an anti-inflammatory functional role through the decreased production of IL-6 and IL-8 by macrophages through polarization. The results from this study partially revealed the effect of IAHA on cytokines and immune cells change in KOA patients, and therapies targeting pathogenic cytokines and immune cells might be used to attenuate the knee joint inflammation and release pain.

**Trial registration:**

ChiCTR2100050133; date registered 17 August 2021.

## Background

Knee osteoarthritis (KOA), as the most common chronic generative joint disease, mainly manifests knee joint pain, functional limitations, and even disability [1]. The exact pathogenesis of KOA remains unclear, but it is considered as an inflammatory disease involving immune cells and their effector cytokines [2]. The interaction between immune cells and cytokines is a dynamic process. The interaction of positive and negative stimuli is complex, often involving multiple immune cells and cytokines [3] However, the role of immune cells and cytokines in the pathogenesis and progression of KOA is still not elucidated [4].

In the management of KOA, current interventions are physical and pharmacological therapies, aiming to relieve pain and improve physical function, but all the interventions can’t stop the disease progression [5]. Intraarticular hyaluronic acid(IAHA)is a standard treatment for KOA patients after failure to education and structured exercise programs, especially those who cannot tolerate the contraindications of Nonsteroidal Anti-Inflammatory Drugs (NSAIDs) or those who still have symptoms like pain or functional limitations after use of NSAIDs, as the European society for clinical and economic aspects of osteoporosis, osteoarthritis and musculoskeletal diseases (ESCEO) and Osteoarthritis Research Society International (OARSI) 2019 guideline-recommended [6–8]. HA plays multiple roles, such as articular cartilage lubrication, analgesic, anti-inflammatory, chondroprotective, by interacting with receptors, enzymes, and many other biomolecules [9]. Studies showed that HA interact with chondrocytes, synoviocytes, osteocytes and immune cells, regulating cell proliferation, differentiation and migration, and effects on immune cells including reduction of motility of lymphocytes and macrophages [10]. It also effects on inflammatory mediators such as reduced levels of prostaglandins, leukotrienes, IL-1 and IL-6, etc. [10, 11]. However, there is still a lot of unclear mechanisms in IAHA treatment.

Previous studies showed that high molecular weight HA (≥ 3000 kDa) displays anti-inflammatory and immunosuppressive properties and had better effect than low molecular weight HA, so High-molecular-weight HA was used in our study [12, 13].

During the treatment of intra-articular injection, it is accessible to get the synovial fluid, which can present the biological processes, to reveal the changes of inflammation [2]. Therefore, a cohort study was built by analyzing the cytokines and immune cells in synovial fluid during multiple follow-ups, aiming to elucidate the effect of hyaluronic acid on cytokines and immune cell change in KOA patients.

## Methods

### Patients

This study was a prospective observational study, approved by the Ethical Committee of Zhongshan Hospital Affiliated with Fudan University (Shanghai, China) (B2021-287). Each patient signed an informed consent form. 50–85 years old patients who had symptomatic KOA associated with chronic pain and were scheduled for IAHA injection at our hospital were included. High-molecular-weight HA was used. Patients with evidence of inflammatory KOA or endocrine disorder were excluded, and those who had knee injections within the preceding 6 months were also excluded. Patients were excluded from whom no knee joint synovial fluid could be withdrawn at the first visit. Before injection at each visit, each patient was assessed the degree of pain with the Numeric Rating Scale (NRS), which was based on an 11-point scale (0–10), and the degree of functional limitations with Lysholm knee score, which contained eight factors rated to produce an overall score on a point scale of 0–100. Kellgren-Lawrence(KL) grade was used to assess the radiographic OA severity.

An experienced rehabilitation physician performed the intra-articular injections according to current standards. The synovial fluid of the knee joint was collected for the measurement of cytokines and immune cell phenotyping. There are 3–5 weekly times of injection in a course in most of the studies, [32935198] so in our study, patients received consecutive injections at weekly intervals, most of them took five times, and some did3-4 treatment. Only patients who had at least three times visits were included to analyze.

### Sample collection and processing

All knee joint synovial fluids of the patients enrolled in this study were collected. 2 mL of synovial fluid of each patient was extracted by the sterile syringe, and then added to an EDTA-anticoagulation tube (BD, Catalog No. 367856), stored at 4℃ in a refrigerator and would be dealt within 24 h. When performing sample processing, 500 × g for 5 min was used to centrifugate the anticoagulation tube. 1 mL of the centrifuged sample suspension was taken out and added to a 1.5 mL Eppendorf tube, stored for cytokine measurements at -20℃ in the refrigerator. The rest was washed twice with 2 mL 1 × PBS solution at 500 × g for 5 min, then the cells in the sample suspension were performed phenotyping in the BD FACSCantoII flow cytometer.

### Cytokine measurements

The cytometric beads assay (CBA) method was used for the cytokine measurements of each sample, and the detection kit was purchased from Beijing Kuangbo Biotechnology Co., Ltd. (Cat. No.: C60011). The cytokines measured in this study included interleukin-1β (IL-1β), IL-2, IL-4, IL-5, IL-6, IL-8, IL-10, IL-12p70, IL-17A, IL-17F, IL-22, interferon-γ (IFN-γ), tumor necrosis factor α (TNF-α), and TNF-β. The tiraes ratio dilution for the mixed standard was used to create the standard curve for each cytokine. 45uL of fluorescent beads were added in each well of the 96-well plate (Merck Millipore, Cat. No.: MSBVN1250), then the liquid was drained off with a suction filter, and 45uL of two-fold diluted sample was added and incubated for 60 min with shaking (approximately 700 rpm) in the dark at room temperature. Each well was washed with 100μL 1 × washing solution (diluted by 2 × washing solution in the kit) for three times, and then 25μL 1 × biotin-labeled antibody mixture was added and incubated for 30 min with shaking (approximately 700 rpm) in the dark at room temperature. Then each well was washed with 100μL 1 × washing solution twice, then added 25μL of phycoerythrin-labeled streptavidin (SA-PE) and incubated for 20 min. After washing twice, 200μL 1 × washing solution was added to each well to resuspend the beads. Then 2000 beads in each well were collected in the BD FACSLyric™ flow cytometer. The analysis of data was in the BD FCAP™ Array Software v3.0.

### Immune cell phenotyping

An antibody panel was established for the sample immune cell phenotyping in the flow cytometer. The following antibodies used to detect the surface antigens were included in the panel: CD16 FITC (BD Bioscience, San Jose, CA, USA, cat. no.:560996), CD11b PE (BD Bioscience, cat. no.:561689), CD33 PE-cy7 (Thermo Fisher, cat. no.:25–0331-82), CD14 APC-H7 (BD Bioscience, cat. no.:560270), CD3 BV421 (BD Bioscience, cat. no.:563797), and CD45 V500 (BD Bioscience, cat. no.:560779). The antibodies mixture was thoroughly mixed with the sample cells in a tube, then incubated for 15 min in the dark at room temperature. 1 mL 1 × lysing solution (diluted by BD FACS™ Lysing Solution 10X Concentrate, cat. no.:349202) was added in each sample to lyse red blood cells and incubated for 10 min in the dark at room temperature. 500 × g for 5 min was used for centrifugation, and the supernatant was discarded after centrifugation, then washed once with 1 × PBS solution. 100μL 1 × PBS solution was added to each tube, and all of the sample cells were collected in the flow cytometer. Side Scatter (SSC)/CD45 was used to gate the CD45-positive leukocytes; SSC/CD33 was used to gate the macrophages and neutrophils; CD14/CD16 was used to distinguish the macrophages M1 and M2 subsets; SSC/CD3 was used to distinguish T cells from lymphocytes.

### Statistical analysis

Data were analyzed with the SPSS software version 20.0 (IBM Corp., Armonk, NY, USA). Descriptive statistics are summarized as means ± standard deviations (SD) for continuous variables. Paired t-test was used to compare the difference between the pre-injection and after-injection. The Pearson correlation method was used for the relationship between cytokines and immune cells. A P value of < 0.05 was considered statistically significant.

## Results

### Patient characteristics and pain relief after injection at visits

Sixteen subjects with KOA who were received hyaluronic acid injection treatment at weekly intervals were included in the study (Table 1). There was a total of 65 injections with 63 samples (an average of 4 times per person). Seven patients (43.75%) received 5 injections, three patients (18.75%) with 4 injections and six patients (37.5%) with 3 injections. Four patients stop the treatment because the effect was not good enough during the first three times, while five stop because the effect was so good that they thought no more treatment should be needed. The mean KL grade was 2.56 ± 0.89. Two patients were on OA stage 1, five on stage 2, seven on stage 3, and two on stage 4, KL grade was not associated with outcome. The mean NRS score for pain before the treatment was 6.06 ± 1.98. After the treatment, 11 of whom (68.75%) showed noticeable pain relief and functional improvement. The baseline of effective group and ineffective group is not statistically different. The NRS score decreased to 2.06 ± 2.67 (*P* = 0.000, paired t-test). The mean Lysholm score before injection was 46.25 ± 18.65. After the treatment, it increased to 71.5 ± 17.27 (*P* = 0.000, paired t-test). Nine patients received the anti-inflammatory drugs while seven did not. We did t-test between this two groups, and no significant difference on the pain relief, cytokines change and immune cells change. During the follow-up of more than three months, the patients' symptoms were similar to those just after treatment, except the symptom of one patient worsened. Pain relief and functional improvement were synchronized, so we mainly directed our follow-up analysis on pain.

**Table 1:** Baseline characteristics of the patients

Characteristic	Total Cohort (*N* = 16)	Effective (*N* = 11)	Ineffective (*N* = 5)
Age-years	68.56 ± 6.88	68.00 ± 8.17	69.80 ± 2.77
Body mass index(kg/m2)	23.34 ± 3.04	23.17 ± 2.93	23.74 ± 3.61
KL grade	2.57 ± 0.94	2.55 ± 0.93	2.60 ± 0.89
NRS score before treatment	6.06 ± 1.98	5.36 ± 1.91	7.60 ± 1.14
Lysholm score before treatment	46.25 ± 18.65	46.72 ± 20.91	45.20 ± 14.46
NRS score after-treatment	2.19 ± 2.43	0.82 ± 1.25	5.20 ± 1.30
Lysholm score after-treatment	71.5 ± 17.27	79.09 ± 10.66	54.80 ± 18.09

**Fig. 1:**
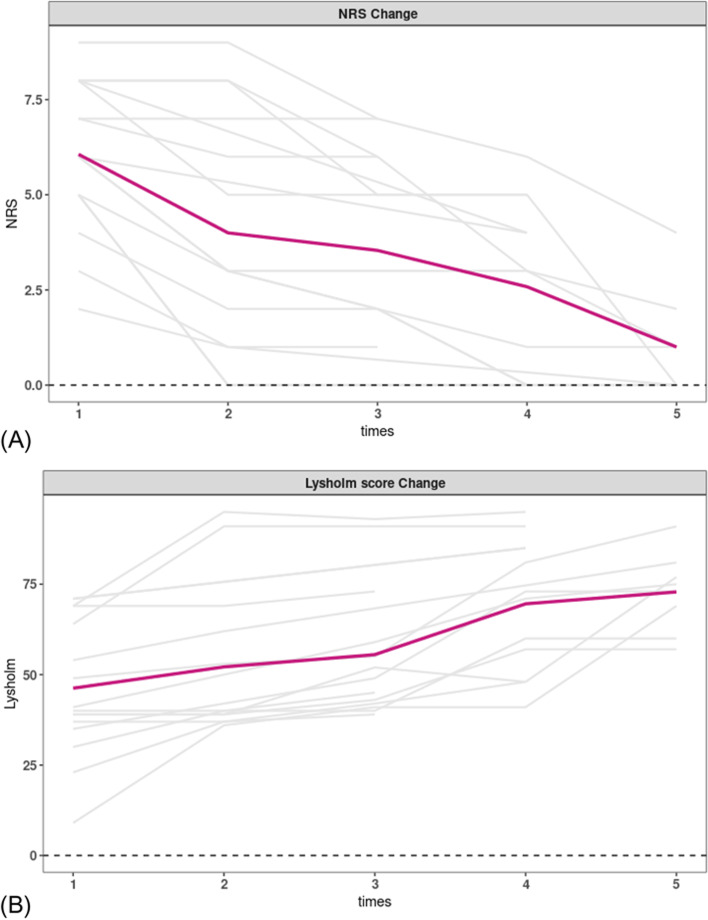
Changes in NRS (**A**) and Lysholm scores (**B**) at follow-up in patients. NRS, Numeric Rating Scale

The changes in NRS scores and Lysholm scores on each visit inpatients are shown in Fig. 1.

### Cytokines change after injection at visits

**Fig. 2:**
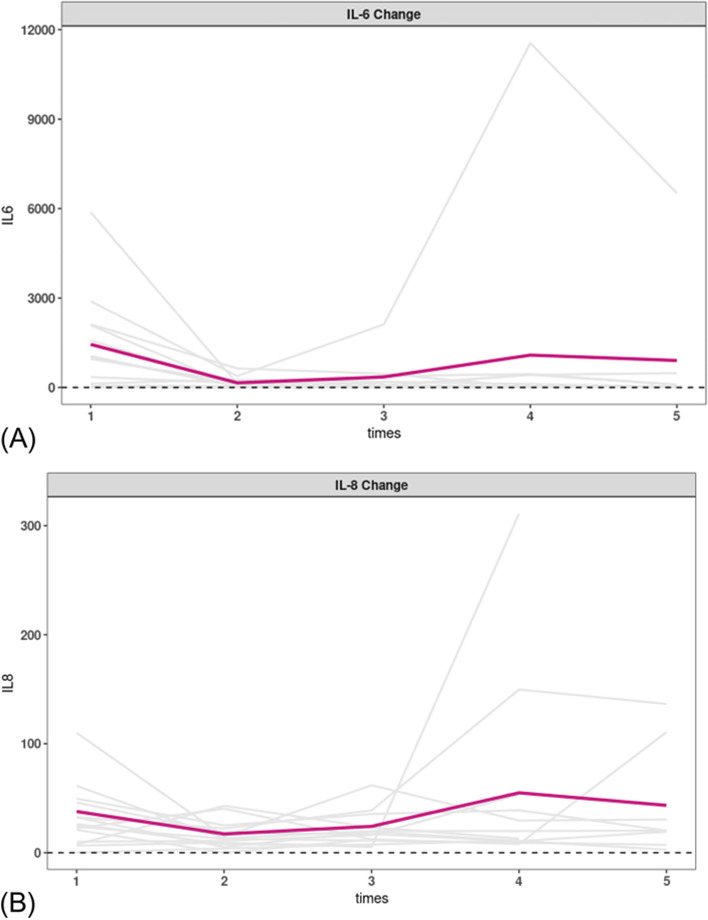
Changes in IL-6 (**A**) and IL-8 (**B**) at follow-up in KOA patients

Fourteen cytokines in the knee joint synovial fluid were measured Their values were near the detection limits (≤ 2 pg/ml), except for the IL-6 and IL-8,so only the changes in IL-6 and IL-8 during follow-up were shown in Fig. 2. The cytokines decreased significantly after the first injection and then tended to be stable. NRS scores were not significantly related to IL-6 or IL-8.

### Cell count change after injection at visits

Using flow cytometry analyses, we distinguished the Leukocytes (CD45bright) and found that the macrophages (SSClow/midCD45brightCD33strongCD14brightCD11bbrightCD16dim/bright), neutrophils (SSCmid/highCD45brightCD33brightCD14negCD11bbrightCD16dim/bright), and T cells (SSClowCD45strongCD3strong) were the three major immune cell populations in the synovial fluid.

**Fig. 3:**
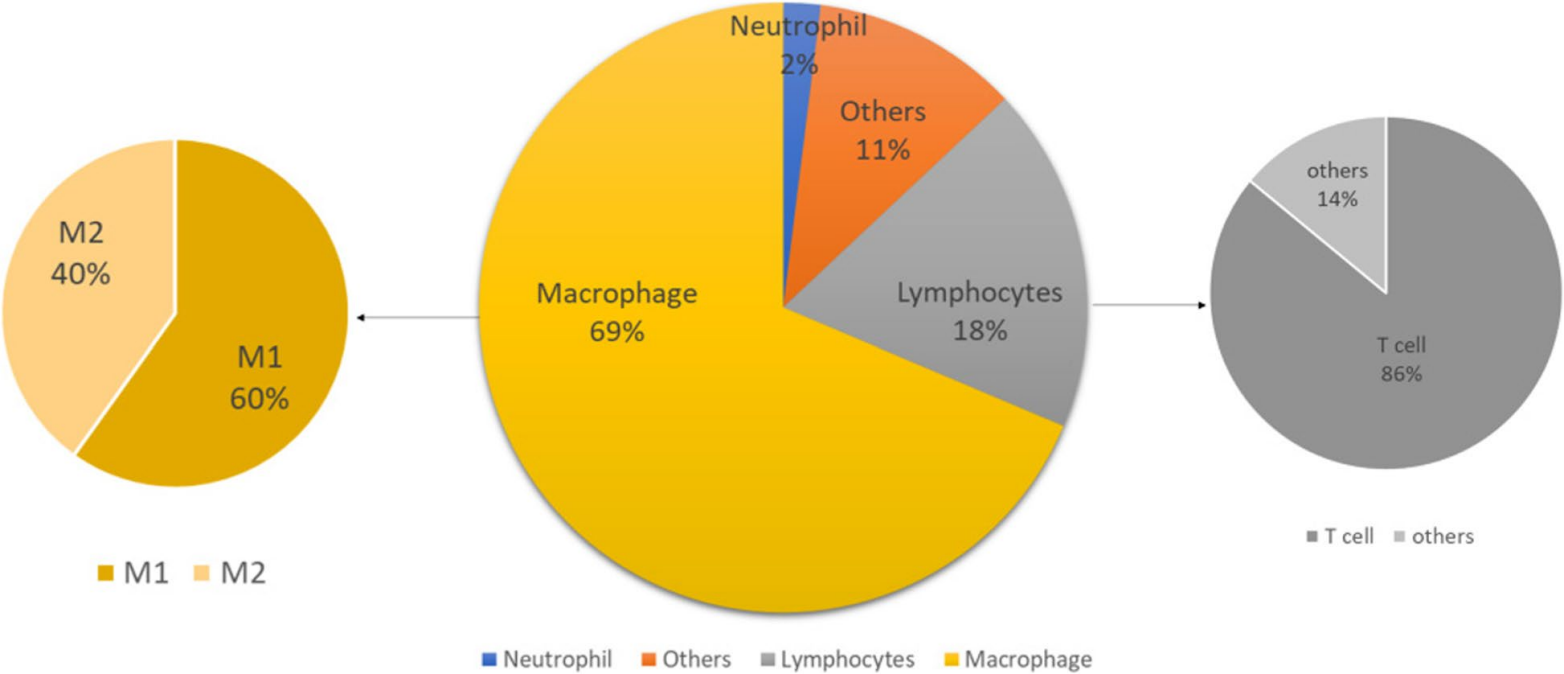
Phenotypic characterization of immune cells by flow cytometry in the synovial fluid of KOA patient

In the synovial fluid of KOA patients, Macrophages were the most abundant immune cell populations, followed by Lymphocytes. Neutrophils were less to count of the total cell population (< 2%). CD14/CD16 was used to distinguish the macrophages M1 subset (CD14brightCD16dim/Bright) and M2 subset (CD14neg/dimCD16neg/dim); SSC/CD3 was used to distinguish T cells from lymphocytes. Results are shown in Fig. 3.

**Fig. 4:**
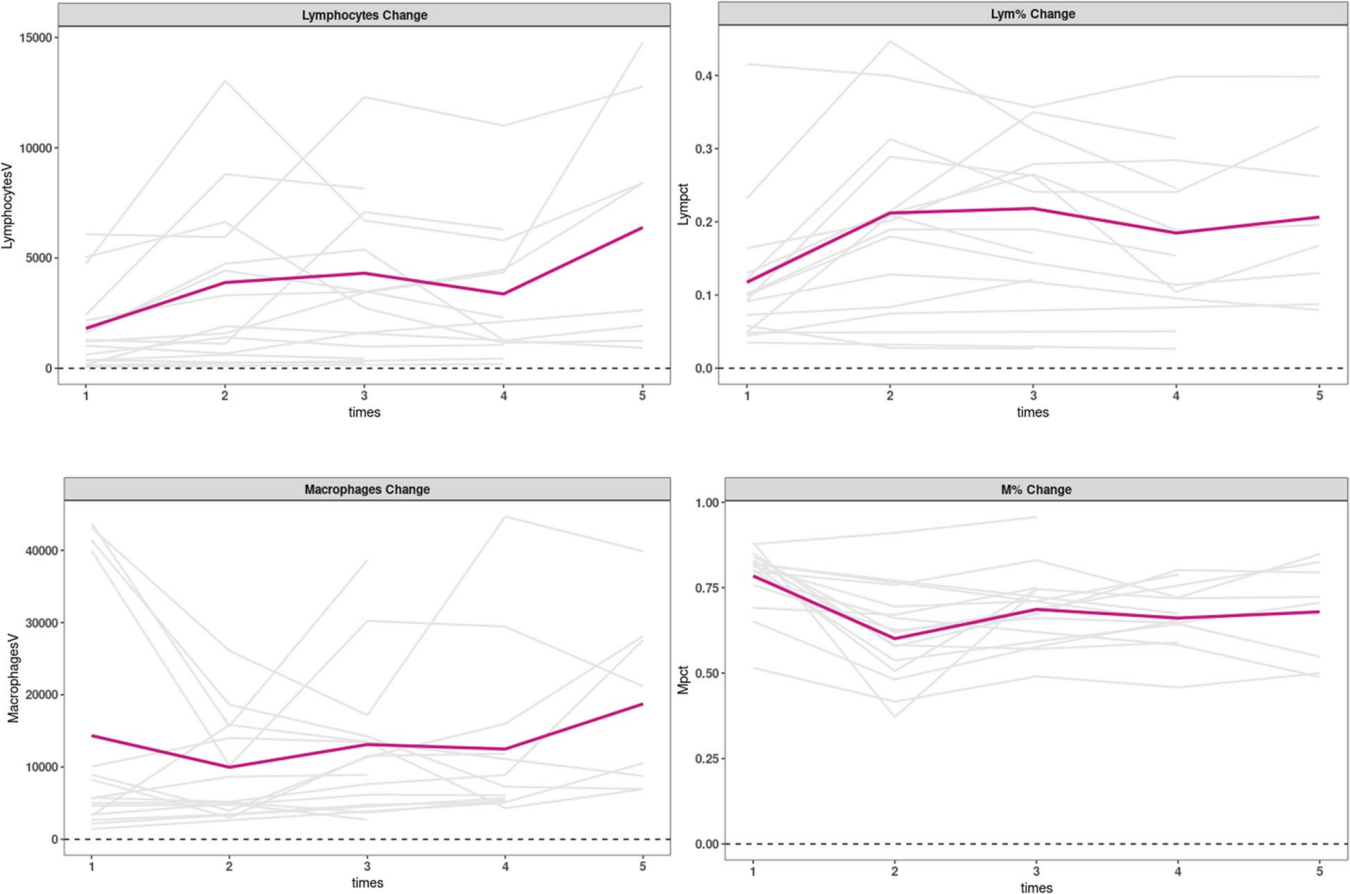
Changes in Immune cells at follow-up in KOA patient

After IAHA injection treatment, Lymphocytes increased a lot, while Macrophages decreased in the early stage, then increased after multiple injections, as Fig. 4 showed.

**Fig. 5:**
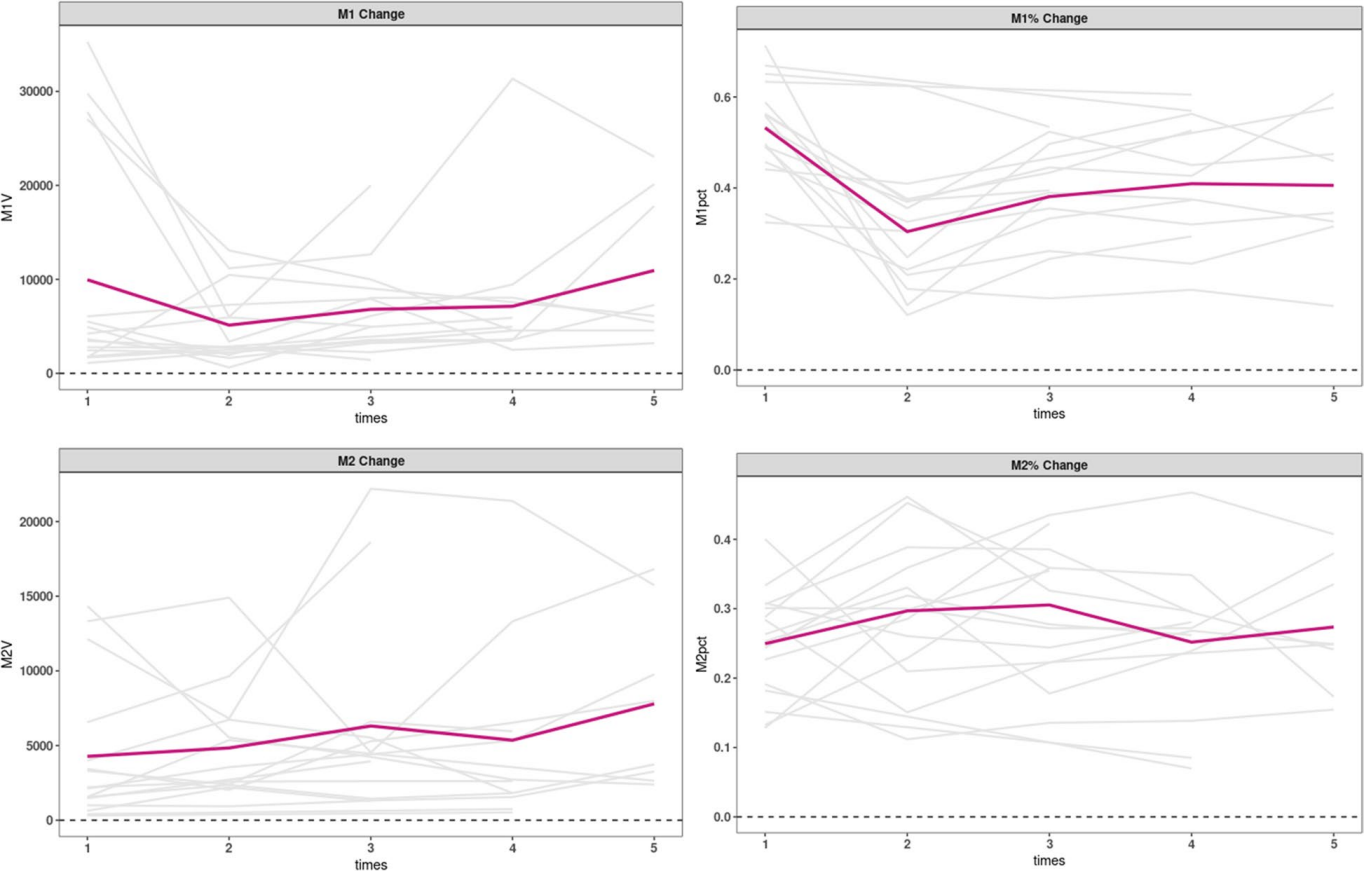
Changes in Macrophages at follow-up in KOA patient

In Macrophages, the proposition of M1 decreased in the early stage, then increased after multiple injections, while M2 increased consistently, as shown in Fig. 5.

**Fig. 6:**
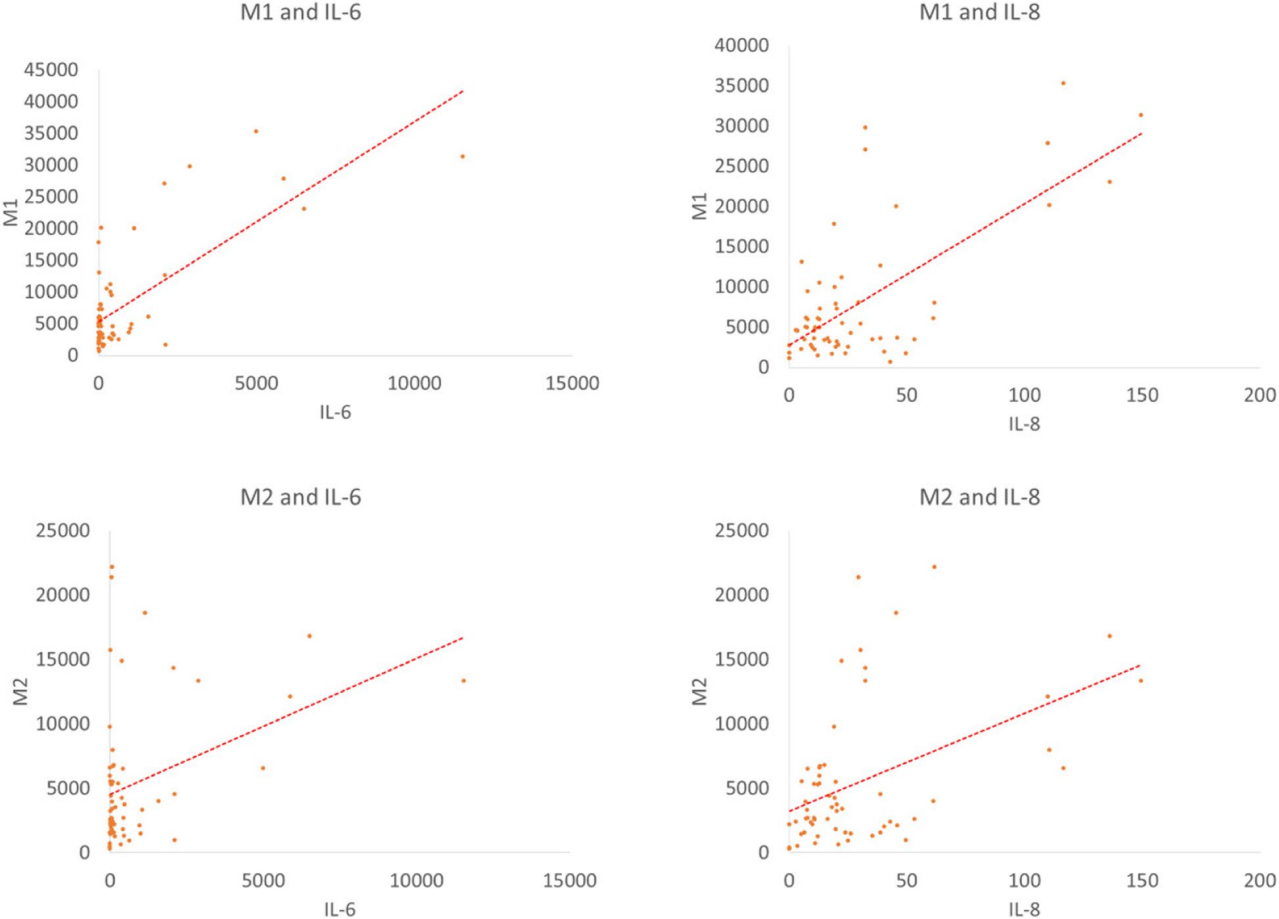
The relationships between Macrophages and cytokines

All samples were included to analysis, and we found that both M1(*p* = 0.000) and M2(*p* = 0.002) were positively associated with IL-6. After removing an extreme value of IL-8, both M1(*p* = 0.000) and M2(*p* = 0.000) were positively associated with IL-8. The relationships are shown in Fig. 6. IL-6 was also positively associated with Neutrophils, but we will not consider its effectiveness because the number of Neutrophils was too small.

## Discussion

IAHA is an alternative treatment for KOA patients and our study showed pain relief and functional improvement in KOA patients who took IAHA treatment.

Previous studies have showed that macrophages and T cells particularly infiltrate OA joints and associated with the increase of cytokines [14]. In this study, we found that the synovial fluid of KOA patients was orchestrated by macrophages (69%) and Lymphocytes (18%), and multiple inflammatory cytokines, especially IL-6 and IL-8. The proportion of Neutrophils was tiny, which was only 2%. Lymphocytes were mainly composed of T cells (86%). In Macrophages, the ratio of M1 to M2 was 6:4. Previous studies had also analyzed the cellular components of SF, and there were differences between these studies. Ming-Feng Hsueh et. showed that in KOA patients who were going to have a knee replacement, SF was mainly orchestrated by macrophages, neutrophils, and T cells, each accounting for about 30% [4]. E. Kriegova et. showed that in KOA patients, that proportion of cells was Lymphocytes (44.8%), Macrophage (14.8%), and Neutrophils (8%) [15]. The difference between those data may be related to the KOA stage of the patient. That study of Ming-Feng Hsueh et. suggested that Neutrophils were involved in the progression of OA, and Neutrophils may be significantly increased in patients with advanced OA [2]. Previous study also confirmed that OA progression is characterized by a distinct influx of inflammatory cells, and the inflammatory pattern differs between different stage of KOA [14]. In our study, patients were relatively early, so the proportion of Neutrophils was less.

Macrophages can generally be divided into pro-inflammatory M1 macrophages and regulatory M2 macrophages. M1 macrophages produce inflammatory cytokines as an integral part of host defense, while M2 macrophages produce anti-inflammatory cytokines [16, 17]. Some studies have shown that a high M1 to M2 ratio was strongly associated with pain and radiographic severity in knee arthritis patients [18, 19]. Our study showed macrophages changes after HA intra-articular injection: M1 decreased at first, then increased slightly, while M2 increased relatively steadily. The significant decrease of M1 after the first treatment was following the law of immunosuppression. The polarization of macrophages from M1 to M2 was associated with pain relief. However, the change of M1 and M2 was not linear. It may suggest that the strict classical subdivision of M1 and M2 macrophages might be questionable. M1 macrophages increased slightly after several injections. Immune tolerance may play a role in this phenomenon [20], for we also found a phenomenon that if the first two injections were not effective, the follow-up effect was also not good.

Abundant pro-inflammatory cytokines were proved to be pathogenic. Studies have reported that up-regulating the expression of IL-6 and IL-8 were potentially involved in OA progression [21, 22]. In this study, IL-6 and IL-8 decreased after the first injection of IAHA, especially IL-6, which was the most variable cytokines. The decrease of IL-6 and IL-8 suggested that IAHA had an anti-inflammatory function. However, the cytokines increased during the progress of injections, which may suggest that one cytokine was implicated in a large number of signal pathways, which was not just about one or two cytokines. This is one of the limitations that only fourteen cytokines were measured in our study.

The number of macrophages, both M1 and M2, were positively associated with IL-6 and IL-8. IL-6 and IL-8 can be synthesized by a variety of cells, including macrophages. Taken together, these findings showed that IAHA might play an influential role in the production of IL-6 and IL-8 by macrophages through polarization. In recent study demonstrated that SF macrophages and Neutrophils were positively associated with IL-6, which showed that IL-6 is generally involved in OA inflammatory responses but may not be representative of a specific cell type [4].

## Conclusion

The synovial fluid of KOA patients was orchestrated by macrophages (69%) and Lymphocytes (18%), and cytokines like IL-6 and IL-8. HA may play an anti-inflammatory functional role through the decreased production of IL-6 and IL-8 by macrophages through polarization. The results from this study partially revealed the effect of IAHA on IAHA treatment in KOA patients, and therapies targeting pathogenic cytokines and immune cells might be used to attenuate the knee joint inflammation and release pain.

## Data Availability

The dataset analysed is not publicly available because data sharing was not part of the original consent and requires institutional approval, but data requests should be submitted to the corresponding author and summary data may be granted following review.

## Abbreviations

IAHA: Intra-articular hyaluronic acid
KOA: Knee osteoarthritis
NSAIDs: Non-Steroidal Anti-Inflammatory Drugs
ESCEO: The European society for clinical and economic aspects of osteoporosis, osteoarthritis and musculoskeletal diseases
OARSI: Osteoarthritis Research Society International
NRS: Numeric Rating Scale
KL: Kellgren-Lawrence
CBA: Cytometric beads assay
IL: Interleukin
IFN: Interferon
TFN: Tumor necrosis factor
SA-PE: Phycoerythrin-labeled streptavidin
